# Differentiation syndrome in patients with acute promyelocytic leukemia

**DOI:** 10.1002/ccr3.6697

**Published:** 2023-01-26

**Authors:** Nusiba H. Elamin, Farah Rashid, Muhammad S. Afana, Honar Cherif, Mohamed A. Yassin

**Affiliations:** ^1^ Department of Internal Medicine Hamad General Hospital, Hamad Medical Corporation Doha Qatar; ^2^ Department of Medical Oncology, Hematology Section, National Center for Cancer Care and Research Hamad Medical Corporation Doha Qatar

**Keywords:** acute leukemia, ATRA syndrome, steroids

## Abstract

A 48‐year‐old male diagnosed with acute promyelocytic leukemia (APL) started on all‐trans‐retinoic acid and arsenic trioxide, developed typical symptoms of differentiation syndrome, and improved dramatically on steroids. Hence, any APL patient started on chemotherapy, needs to be monitored closely for developing differentiation syndrome and to start steroid upon suspicion.

## Graphical abstract

Differentiation syndrome should be suspected and considered on the top of the differential diagnoses whenever you encounter a patient with acute promyelocytic leukemia who deteriorates after starting chemotherapy with ATRA/ATO and prompt management with steroids is crucial in order to avoid this fatal complication.

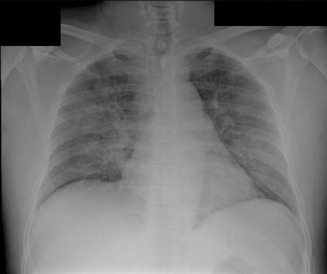

## 1 CASE PRESENTATION

**FIGURE 1:**
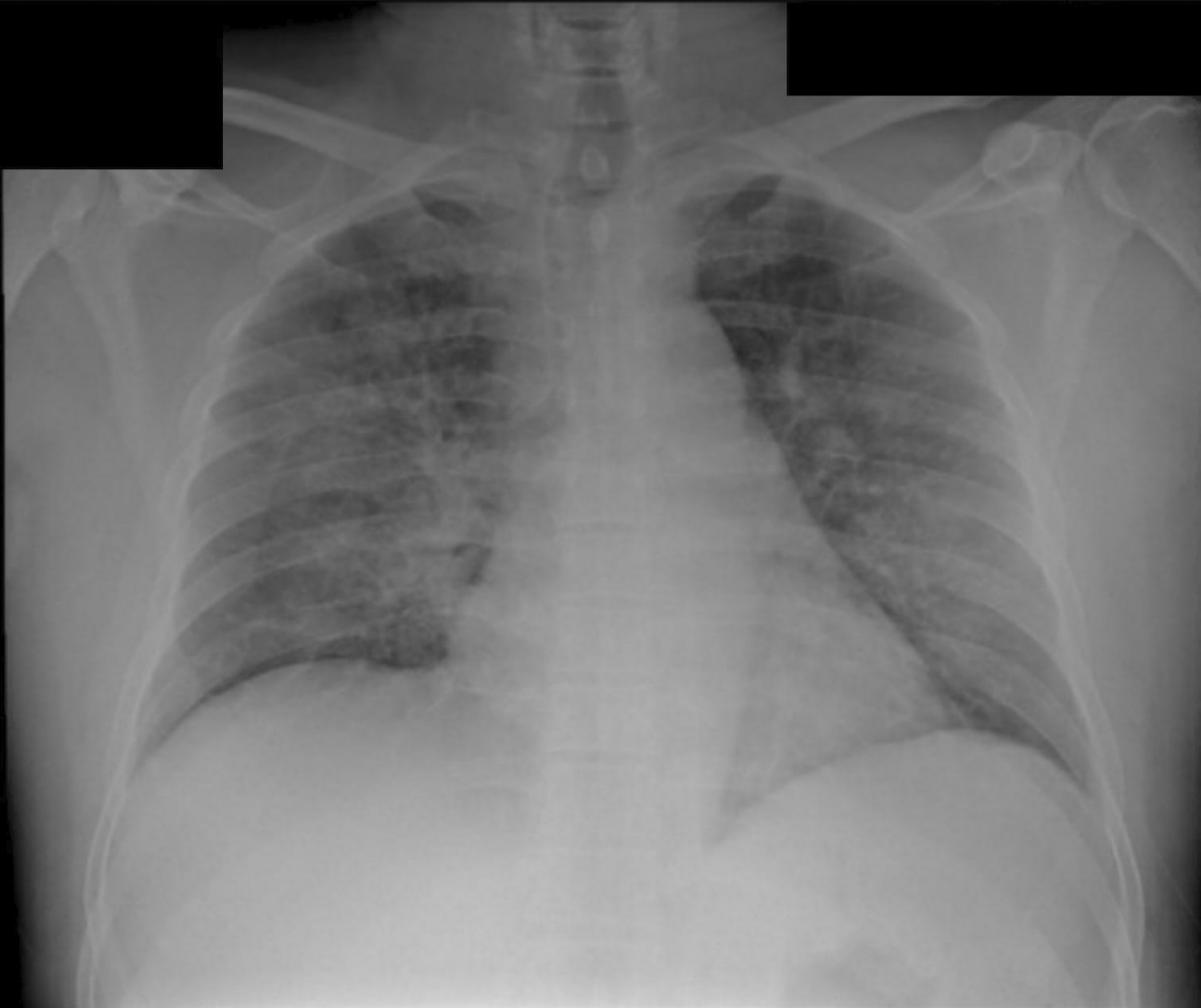
Bilateral opacities extending laterally in a fan shape from the hilum

**FIGURE 2:**
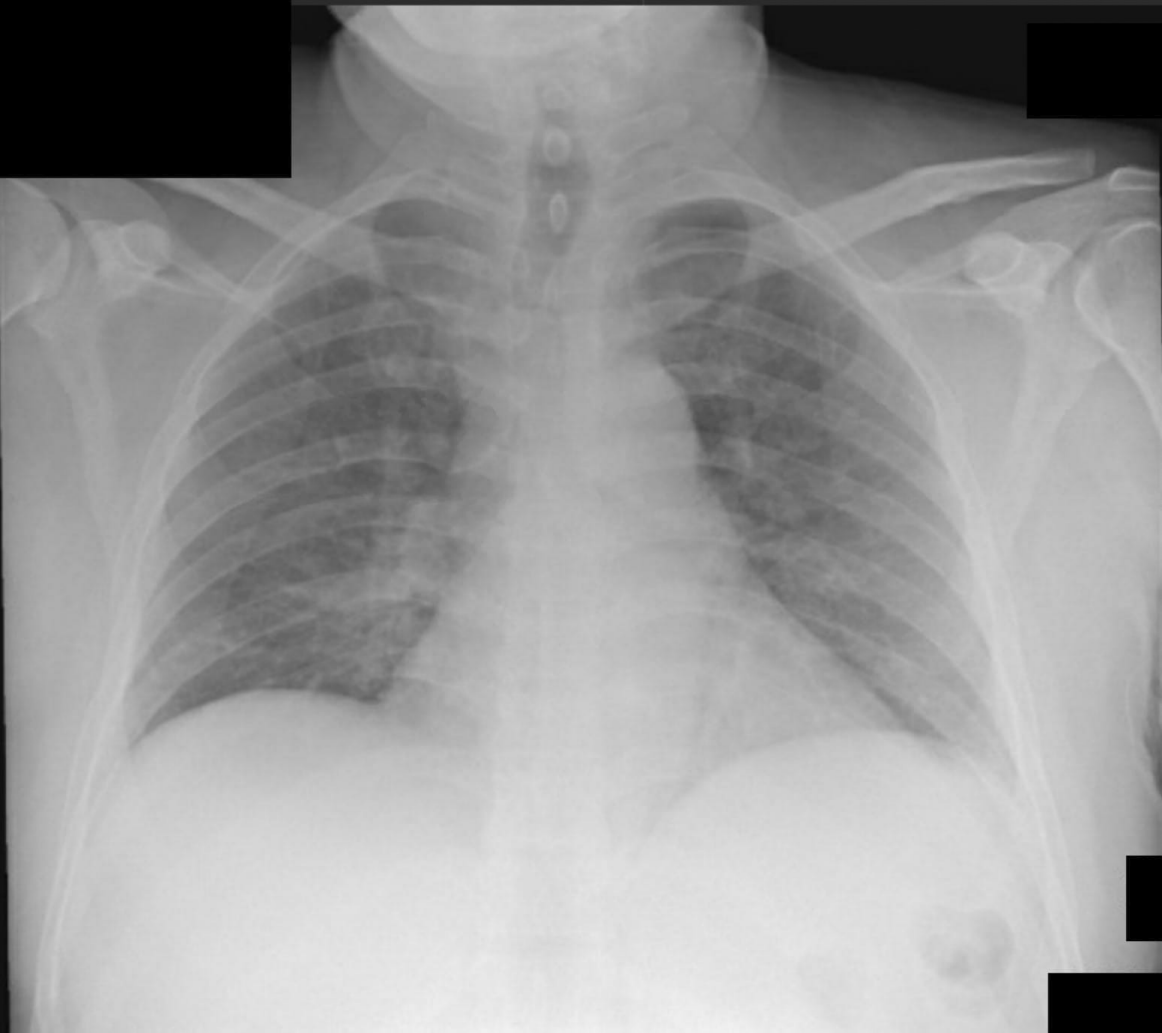
Resolution of the bilateral opacities as complare to previous chest X‐ray

A 48‐year‐old male diagnosed with APL started on all‐trans‐retinoic acid (ATRA) and arsenic trioxide (ATO) as per protocol,^1^ on Day 2 he started to have fever, dyspnea, and hemoptysis. Chest X‐ray (CXR) showed bilateral infiltration (Figure 1), and the patient was started on dexamethasone, based on suspicion of differentiation syndrome. The patient improved significantly within 24 h, and follow‐up CXR showed resolution of infiltrates (Figure 2). Diagnosis of differentiation syndrome requires 3 or more of clinical features: fever>38 c, weight gain >5 kg, hypotension, dyspnea, radiographic opacities, pleural or pericardial effusion, and acute renal failure.^2^

## AUTHOR CONTRIBUTIONS

NE was involved in the literature review and initial manuscript writing. FR contributed to data collection and study conception. MA and HC were involved in patient clinical care and drafting of the manuscript. MY was involved in the literature search, study conception, and drafting and revision of the manuscript. All authors critically reviewed the article, gave final approval of the version to be published and agreed to be accountable for all aspects of the work.

## FUNDING INFORMATION

Open Access funding provided by the Qatar National Library.

## CONFLICT OF INTEREST

The authors declare that there was conflict no of interest regarding the publication of this case report.

## CONSENT

Written informed consent was obtained from the patient to publish this report in accordance with the journal's patient consent policy.

## Data Availability

The datasets used and/or analyzed during the current study are available from the corresponding author on reasonable request.
